# Impact of COVID-19 in patients with multiple myeloma based on a global data network

**DOI:** 10.1038/s41408-021-00588-z

**Published:** 2021-12-10

**Authors:** J. Martinez-Lopez, G. Hernandez-Ibarburu, R. Alonso, J. M. Sanchez-Pina, I. Zamanillo, N. Lopez-Muñoz, Rodrigo Iñiguez, C. Cuellar, M. Calbacho, M. L. Paciello, R. Ayala, N. García-Barrio, D. Perez-Rey, L. Meloni, J. Cruz, M. Pedrera-Jiménez, P. Serrano-Balazote, J. de la Cruz

**Affiliations:** 1 Hematology Department, Hospital 12 de Octubre, Complutense University, CNIO, Madrid, Spain; 2 Biomedical Informatics Group, Universidad Politécnica de Madrid, Madrid, Spain; 3 Data Science Group, Hospital 12 de Octubre, Madrid, Spain; 4 TriNetX, LLC, Cambridge, MA USA; 5 Research Institute imas12, Hospital 12 de Octubre, Madrid, Spain

**Keywords:** Epidemiology, Risk factors

## Abstract

The COVID-19 pandemic has represented a major cause of morbidity/mortality worldwide, overstressing health systems. Multiple myeloma (MM) patients show an increased risk for infections and they are expected to be particularly vulnerable to SARS-CoV-2 infection. Here we have obtained a comprehensive picture of the impact of COVID-19 in MM patients on a local and a global scale using a federated data research network (TriNetX) that provided access to Electronic Medical Records (EMR) from Health Care Organizations (HCO) all over the world. Through propensity score matched analyses we found that the number of new diagnoses of MM was reduced in 2020 compared to 2019 (RR 0.86, 95%CI 0.76–0.96) and the survival of newly diagnosed MM cases decreased similarly (HR 0.61, 0.38–0.81). MM patients showed higher risk of SARS-CoV-2 infection (RR 2.09, 1.58–2.76) and a higher excess mortality in 2020 (difference in excess mortality 9%, 4.4–13.2) than non-MM patients. By interrogating large EMR datasets from HCO in Europe and globally, we confirmed that MM patients have been more severely impacted by COVID-19 pandemic than non-MM patients. This study highlights the necessity of extending preventive measures worlwide to protect vulnerable patients from SARS-CoV-2 infection by promoting social distancing and an intensive vaccination strategies.

## Introduction

Since its outbreak at the end of 2019, the coronavirus disease 2019 (COVID-19) has overstretched National Health Systems (NHS) worldwide and had a profound impact on healthcare quality and access [1–3]. The excess mortality of 2020 compared to the previous years observed in many countries has been attributed to COVID-19 [4, 5], whose effects have been particularly devastating in the oncologic population [6–12].

Multiple myeloma (MM) patients are known to have variable degrees of multifactorial immunodeficiency related to the disease itself and the administered therapies. Therefore the risk of infection is increased in MM patients, and it is a major cause of morbidity and mortality [13–16]. In particular, recent studies have shown that viruses represent a frequent etiology of infections in MM patients [17, 18]. Multicenter and international clinical studies have documented that patients with MM are a vulnerable population at high risk of hospitalization and death following a COVID-19 infection [19–23]. However, there are no data evaluating whether MM patients specifically are at increased risk of infection by SARS-CoV-2 compared with the overall population.

We hypothesized that the COVID-19 pandemic might have decreased or delayed the diagnosis of MM for many patients, in comparison with the pre-COVID-19 era [24, 25], in the context of overburdened NHS, movement restrictions, and other measures implemented to control the spread of the infection. Additionally, we tried to confirm that MM patients were disproportionately impacted by COVID-19 compared with non-MM patients in terms of incidence of COVID-19 infection and survival outcomes.

Since geographical differences may exist between countries such as the incidence of COVID-19, the organization of healthcare delivery, the availability of health resources, the government strategy to combat the pandemic, and many other factors, it seemed appropriate to draw on a global data network to obtain a more accurate and comprehensive picture of the impact of COVID-19 in MM patients. Large network data platforms based on electronic medical records (EMRs) provide capabilities for comparing cohorts across time periods and clinical profiles. For this study, we used TriNetX, a global health research network, to test our hypothesis in an attempt to overcome some of the constraints of previous studies which used local or regional data sources. This platform has previously been shown to be a useful tool for answering research questions in different settings of diseases [26–30].

Thus, the general objective of this study was to provide comparative data on a local, regional and global scale on the impact of COVID-19 on MM patients. The specific aims of our study were to compare the occurrence of new MM diagnoses and the survival of MM patients in 2020 and 2019, and to compare the proportion of COVID-19 cases and the excess mortality in MM and non-MM patients.

## Methods

### Database network and patients selection

This study was conducted with data obtained from TriNetX, LLC (“TriNetX”) a global federated health research network that provides access to EMRs from healthcare organizations (“HCOs”) all over the world. TriNetX provides access to data containing diagnoses, procedures, medications, laboratory values, genomic information from approximately 90 000 MM patients from over 66 HCOs from the 68 that are part of the network. The analyses were conducted utilizing three networks to confirm our hypotheses: the Hospital 12 de Octubre network (H12O), with 930 000 patients; the TriNetX EMEA Collaborative Network (“EMEA”), with 9,800,000 patients from 15 HCOs (including Hospital 12 de Octubre); and the TriNetX Global Collaborative Network (“Global”), with 82 000 000 patients from 68 Healthcare Organizations (including EMEA HCOs). All data collection, processing, and transmission were performed in compliance with all Data Protection laws applicable to the contributing HCOs, including the EU Data Protection Law Regulation 2016/679, the General Data Protection Regulation on the protection of natural persons with regard to the processing of personal data and the Health Insurance Portability and Accountability Act (“HIPAA”), the US federal law which protects the privacy and security of healthcare data. The TriNetX EMEA and Global Collaborative Networks are distributed networks, and analytics are performed on anonymized or pseudonymized/de-idenfied (per HIPAA) data housed at the HCOs, with only aggregate results being returned to the TriNetX platform. Individual personal data does not leave the HCO. TriNetX is ISO 27001:2013 certified and maintains a robust IT security program that protects both personal data and health care data.

MM patients were identified by the presence of the parent ICD-10-CM code for MM (C90.0) or any of the specific codes (C90.00, C90.01 and C90.02) in their EMR. In addition, we included patients whose MM was miscoded initially as monoclonal gammopathy by identifying patients who had the diagnosis code D47.2 (monoclonal gammopathy) and had been treated with one of the following MM treatments: thalidomide, bortezomib, lenalidomide, daratumumab, melphalan, ixazomib or carfilzomib. For the overall survival analysis, the first day of MM was either the diagnosis or the start date of the treatment, whatever happened first. The infection of COVID-19 was identified as either a positive PCR test or an ICD-10-CM diagnosis of COVID-19 in 2020, ICD codes U07.1 (COVID-19, Virus identified), U07.2 (COVID-19, Virus not identified), or B97.29 (Other coronavirus as the cause of diseases classified elsewhere). Finally, included patients were ≥25 years years old.

### Patient flowchart and characteristics

A total of 855 patients with MM criteria were identified in H12O network, 7265 in EMEA, and 83,550 in Global. Control cohorts of non-MM patients were identified by propensity score matching 1:1 on age and gender from a pool of individuals without any diagnoses of MM or monoclonal gammopathy and without any record of receipt of MM treatments (Table 1). For the non-MM patients, the cohort was limited to patients thathad a hospital visit in the last 5 years. The three networks showed a higher probability (*p* < 0.005) of hypertension, other forms of heart diseases (different from ischemic heart diseases), chronic kidney disease and diseases of the musculoskeletal system, disorders of bone density for patients with MM. MM patients in the EMEA and Global comparisons also showed a significantly higher probability of diabetes mellitus, other dorsopathies, injuries and lower mean value of glomerular filtration rate (MDRD) compared with non-MM patients. In addition, a significantly higher percentage of MM patients in the global cohort had ischemic heart diseases.

**Table 1:** Characteristics of Multiple Myeloma (MM) cohorts in H12O, EMEA and Global data networks, compared with non-MM cohorts before and after propensity score matching (PSM) on age and gender.

Demographics							
Cohort		Characteristic	MM N(%)/mean (STD)	non-MM N(%)/mean (STD)		*P* value (Standard mean difference**)	
				Before PSM	After PSM	Before PSM	After PSM
H12O	**662,545**	Current Age	71.26 (12.23)	43.06 (25.56)	71.26 (12.26)	<0.001 (1.40)	0.99(0)
		Male	442 (51.70%)	316,079 (47.77%)	443 (51.81%)	0.021 (0.07)	0.96(0)
		Female	413 (48.30%)	345,611 (52.23%)	412 (48.19%)	0.021 (0.07)	0.96 (0)
EMEA	**6,446,411**	Current Age	73.36 (10.71)	50.51 (24.84)	73.36 (10.71)	<0.001 (1.19)	1 (0)
		Male	4282 (58.94%)	3,021,909 (46.87%)	4282 (58.94%)	<0.001 (0.24)	1 (0)
		Female	2983 (41.06%)	3,417,237 (53.01%)	2983 (41.06%)	<0.001 (0.24)	1 (0)
Global	7,748,356*	Current Age	69.68 (11.83)	57.81 (17.91)	69.68 (11.83)	<0.001 (0.78)	1 (0)
		Male	45,803 (54.82%)	3,384,316 (43.99%)	45,803 (54.82%)	<0.001 (0.21)	1 (0)
		Female	37,350 (44.70%)	4,280,887 (55.65%)	37,350 (44.70%)	<0.001 (0.22)	1 (0)

Laboratory				
		MM	non-MM	*p*
		mean (std)	mean (std)	
H12O	GFR (MDRD)	74.13 (39.82)	77.19 (26.78)	0.12
	Hemoglobin in blood (g/dL)	11.71 (2.29)	13.78 (2.09)	<0.001
EMEA	GFR (MDRD)	68.35 (37.72)	75.79 (28.78)	<0.001
	Hemoglobin in blood (g/dL)	11.12 (2.26)	13.00 (2.23)	<0.001
Global	GFR (MDRD)	65.42 (33.80)	73.29 (27.68)	<0.001
	Hemoglobin in blood (g/dL)	11.24 (2.47)	12.84 (2.52)	<0.001

Diagnoses					
Cohort	Characteristic name	MM	non-MM	Risk ratio*** (95% CI)	*p* value
H12O	Hypertension	333 (38.95%)	241 (28.19%)	1.38 (1.21,1.58)	<0.001
	Ischemic heart diseases	80 (9.36%)	76 (8.89%)	1.05 (0.78,1.42)	0.737
	Other forms of heart disease	250 (29.24%)	159 (18.60%)	1.57 (1.32,1.87)	<0.001
	Diabetes mellitus	132 (15.44%)	125 (14.62%)	1.06 (0.84,1.32)	0.636
	Chronic kidney disease	114 (13.33%)	52 (6.082%)	2.19 (1.60,3.00)	<0.001
	Injuries	134 (15.673%)	116 (13.567%)	1.15 (0.91,1.44)	0.218
	Other dorsopathies	76 (8.89%)	49 (5.73%)	1.61 (1.11,2.33)	0.012
	Disorders of bone density and structure	143 (16.73%)	42 (4.91%)	3.41 (2.45,4.74)	<0.001
	Chronic lower respiratory diseases	91 (10.64%)	67 (7.84%)	1.36 (1.01,1.84)	0.045
EMEA	Hypertension	2933 (40.37%)	2339 (32.20%)	1.25 (1.20,1.31)	<0.001
	Ischemic heart diseases	1174 (16.16%)	1235 (17.00%)	0.95 (0.88,1.02)	0.174
	Other forms of heart disease	2306 (31.74%)	1486 (20.45%)	1.55 (1.47,1.64)	<0.001
	Diabetes mellitus	1097 (15.10%)	950 (13.08%)	1.16 (1.07,1.25)	<0.001
	Chronic kidney disease	1658 (22.82%)	465 (6.40%)	3.57 (3.23,3.93)	<0.001
	Injuries	1125 (15.46%)	967 (13.287%)	1.17 (1.07,1.26)	<0.001
	Other dorsopathies	1139 (15.65%)	414 (5.688%)	2.75 (2.46,3.06)	<0.001
	Disorders of bone density and structure	978 (13.44%)	222 (3.05%)	4.41 (3.82,5.08)	<0.001
	Chronic lower respiratory diseases	1070 (14.73%)	823 (11.33%)	1.30 (1.19,1.42)	<0.001
Global	Hypertension	45,193 (54.09%)	38,144 (45.65%)	1.19 (1.17,1.20)	<0.001
	Ischemic heart diseases	18,146 (21.72%)	14,505 (17.36%)	1.25 (1.23,1.28)	<0.001
	Other forms of heart disease	36,929 (44.20%)	22,214 (26.59%)	1.66 (1.64,1.69)	<0.001
	Diabetes mellitus	19,101 (22.86%)	17,327 (20.74%)	1.10 (1.08,1.12)	<0.001
	Chronic kidney disease	22,430 (26.85%)	9372 (11.22%)	2.39 (2.34,2.45)	<0.001
	Injuries	25,601 (30.64%)	19,990 (23.92%)	1.41 (1.38,1.43)	<0.001
	Other dorsopathies	31,534 (37.74%)	19,502 (23.34%)	1.62 (1.59,1.64)	<0.001
	Disorders of bone density and structure	20,586 (24.64%)	8626 (10.32%)	2.39 (2.76,2.92)	<0.001
	Chronic lower respiratory diseases	16,925 (20.26%)	13,625 (16.31%)	1.24 (1.22,1.27)	<0.001

*Subsample of the cohort due to computational limitations.

**Fig. 1:**
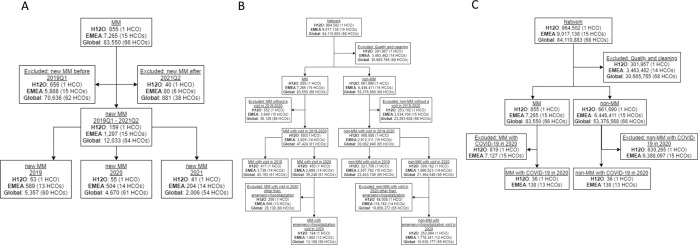
Flow chart of the study. **A** Diagram of the cohorts used for the analysis of newly diagnosed multiple myeloma (MM) cases in 2019 and 2020. **B** Diagram of the cohorts used for the analysis of COVID-19 cases in MM and non-MM patients. **C** Diagram of the cohorts used for the COVID-19 analysis of mortality analysis (MM vs non-MM) between the first and the second half of 2020.

**Fig. 2:**
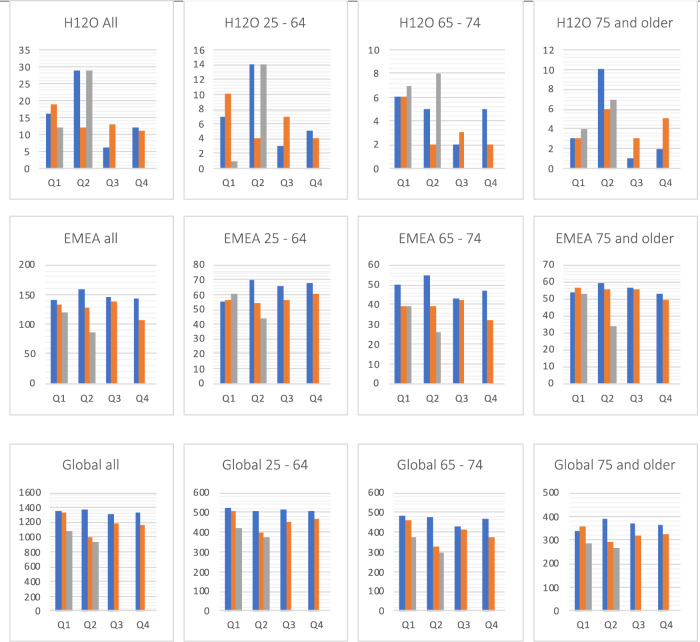
New MM cases by year. New cases of MM by data network, age group and quarters of 2019 (blue), 2020 (orange) and 2021 (gray).

**Fig. 3:**
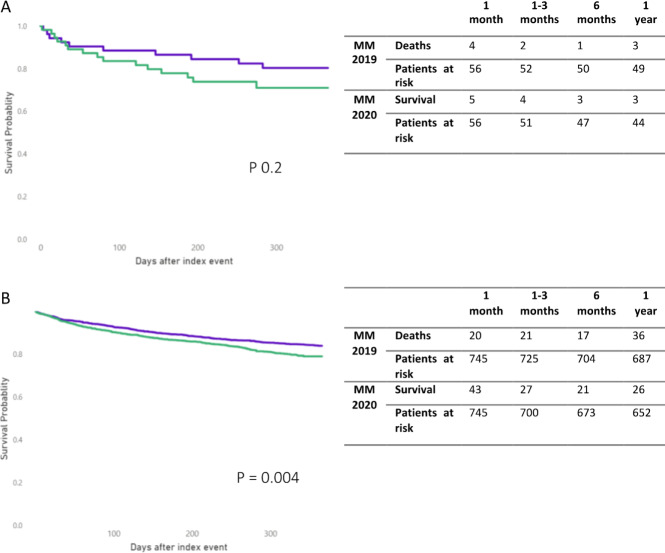
MM survival by year. **A** KM plots comparing OS after the first year of MM diagnosis of patients diagnosed in 2019 (purple) and in 2020 (green), with H12O data. **B** KM plots comparing OS after the first year of MM diagnosis of patients diagnosed in 2019 (purple) and in 2020 (green), with EMEA network.

The MM cohort in each of the networks was further stratified to run the different analyses. One set of sub-cohorts included the patients with a new diagnosis of MM from 2019Q1 to 2021Q2 (Fig. 1A). They were used to evaluate the impact of the COVID-19 pandemic in the number of new MM diagnoses (Fig. 2) and in the overall survival (OS) of newly diagnosed MM patients (Fig. 3). Another set ofsub-cohorts included MM and non-MM patients that had an emergency visit or hospitalization in 2019 or 2020 (Fig. 1B); they were used to assess the incidence of COVID-19 and the excess mortality in 2020. A third set of cohorts was built with COVID-19 patients, MM and non-MM, to analyse the evolution of the OS in the period of January to May, 2020, compared to the period of June to December 2020 (Fig. 1C). In all settings, non-MM cohorts included patients with sufficient information in their EMR to run the analysis: patients without any diagnosis or with an EMR trajectory shorter than 3 months were excluded.

### Statistical analysis

All analyses were generated with TriNetX platform software (TriNetX, Cambridge, MA) in August of 2021 [31]. We compared the incidence (new cases) of MM diagnosis and the survival of MM patients in 2020 and 2019. We also compared the incidence (case count) of COVID-19 and the excess mortality of MM and non-MM patients. Finally, we compared the survival of COVID-19 MM and non-MM cases over two time periods in 2020.

The number of new cases of MM and of COVID-19 cases were compared with risk ratios and 95% confidence intervals (95% CI). Kaplan-Meier analysis was used to estimate survival probabilities, and the difference between groups was tested using the log-rank test and quantified with hazard ratios (95% CI), calculated with TriNetX Analytics features. All the cohorts were propensity score-matched on age and gender. For the survival analysis of MM and non-MM patients and the comparisons between 2020 and 2019, the cohorts were also matched on mortality risk factors for COVID-19 patients: I10-I16 (Hypertensive diseases), E08-E13 (Diabetes mellitus), N18 (Chronic kidney disease), I30-I52 (Other forms of heart disease), M00-M99 (Diseases of the musculoskeletal system and connective tissue), J40-J47 (Chronic lower respiratory diseases) and I20-I25 (Ischemic heart diseases) [32–34]. Global was not used in the survival analysis because of network data limitations on out-of-hospital mortality.

## Results

### New diagnosis of MM cases over time

The total number of new MM patients between 2019Q1 and 2021Q2 was 159 in H12O, 1 297 in EMEA and 12 033 in Global network (Fig. 2). The number of new MM diagnoses was lower in 2020 than in 2019: in H12O, 55 cases vs 63 with a risk ratio (RR) of 0.87 (95% CI 0.62–1.24), *p* = 0.44; EMEA, 504 vs 589, RR = 0.86 (0.76–0.96), *p* = 0.008; and Global network, 4670 vs 5357, RR = 0.87 (0.84–0.91), *p* < 0.001.

### COVID-19 cases in MM and non-MM patients

Then, we analyzed patients that had a hospital visit in 2019 to assess if MM patients had a higher incidence of COVID-19 than subjects without diagnosis of MM. After propensity score matching, the H12O cohort had a total number of 417 patients, mean age of 67.4 years and 196 (47%) males; EMEA had a total number of 3738 patients, mean age of 69.4 and 2119 (57%) males, and Global had a total number of 43 192 patients, mean age of 66.5 and 22,819 (52%) males. In the three cohorts, the likelihood of being diagnosed with COVID-19 was higher in MM than in non-MM patients: 38 (9.11%) vs 29 (6.95%), risk ratio 1.31 (0.82–2.08), *p* = 0.25 with H12O data; 148 (3.96%) vs 71 (1.90%), risk ratio 2.09 (1.58–2.76), *p* < 0.001 with EMEA network; and 2 018 (4.67%) vs 1174 (2.718%), risk ratio 1.72 (1.60–1.85) *p* < 0.001 with Global network.

### COVID-19 impact on survival of MM patients

We also explored the impact of COVID-19 in the survival of newly diagnosed MM patients in H12O and EMEA networks. Patients newly diagnosed with MM in 2019 (MM 2019) were propensity score matched with patients diagnosed in 2020 (MM 2020) on age and gender. After the matching, each cohort had 56 patients, mean age of 67 and a male proportion of 57.90% (MM 2019) and 61.40% (MM 2020) in H12O; 745 patients and mean age of 71 and a male proportion of 61.33% (MM 2019) and 60.93% (MM 2020) in EMEA. Patients diagnosed in 2019 had a higher survival probability one year after the diagnosis than patients diagnosed in 2020: H12O, 82.46% (46) vs 73.68%, (41) hazard ratio 0.64 (0.29, 1.43), *p* = 0.27; EMEA, 87.38% (651) vs 84.50% (628), hazard ratio 0.61 (0.38–0.81), *p* = 0.004, Fig. 3.

We then estimated the excess mortality (2020 vs 2019) in MM and non-MM patients. The balanced cohorts in H12O included 192 MM patients with a 2020 excess mortality of 41%: 32 (16.67%) from the 2019 cohort and 45 (23.44%) from 2020 died. The H12O non-MM balanced cohorts included 134,344 patients with a 2020 excess mortality of 27%: 3 797 (2.83%) died within a year after an emergency or hospitalization visit in 2019 while 4 808 (3.58%) died in 2020. In EMEA, balanced cohorts included 1 962 MM patients with an excess mortality of 16%: 287 (14.63%) died within one year after the hospitalization or emergency visit in 2019 and 332 (16.92%) in 2020. In the non-MM cohorts (*n* = 946,886), excess mortality was 7%: 37 047 (3.91%) died after the 2019 visit and 39,581 (4.18%) after the 2020 visit. Excess mortality in 2020 in both data networks was higher in MM than in non-MM patients: the difference in excess mortality was 14% (−4.7 to 32.6, *p* = 0.1) in H12O and 9% (4.4 to 13.2, *p* < 0.001) in EMEA.

The final analysis compared the survival of COVID-19 MM and non-MM patients diagnosed in two periods, January to May 2020 (first period) and June to December 2020 (second period) (Fig. 1). After balancing cohorts with propensity score matching, the analysis showed no statistically significant differences for the survival of COVID-19 MM patients: in H12O, 84.6% (11) vs 76.9%, (10) hazard ratio 0.62 (0.10–3.73) *p* = 0.6; in EMEA, 72.2% (39) vs 64.8%, (35) hazard ratio 0.73 (0.37–1.43), *p* = 0.35. On the contrary, the survival of COVID-19 non-MM patients increased in the second period: 89.41% (703) vs 96.21% (252), hazard ratio 2.64 (2.29–3.05), *p* < 0.001 in H12O; 88.10% (2059) vs 90.94% (1569), hazard ratio 1.25 (1.17–1.34), *p* < 0.001 in EMEA.

## Discussion

In this real-world data analysis, we showed that diagnosis of new MM cases has decreased during the COVID-19 pandemic at a local and global level. Similarly, the survival of MM patients has decreased in 2020 compared to 2019. Our analysis suggests that MM patients have a higher risk of contracting SARS-CoV-2 infection than non-MM patient population. We found also that the excess mortality in 2020 was higher for MM patients than for non-MM patients. These results confirmed the remarkable impact of the COVID-19 pandemic in the management and outcomes of MM patients.

The COVID-19 pandemic has overloaded our health systems globally; this resulted in a lack of attention to other pathologies that require very specialized diagnostic and therapeutic tools. Some authors hypothesized that the number of newly diagnosed cancer patients has been reduced during pandemic; however, this is the first time in the MM setting that this fact has been demonstrated in a large international multi-site studies with multi-institutional and multi-national patients [9, 24, 25, 28, 35–37]. Our results could mean that globally around 15% of MM patients have not been diagnosed on time, or they have died because of the severe consequences on our health systems and patients. This is consistent with previous analysis on other cancers [28, 29, 38, 39].

There is little information about whether cancer patients have an increased probability of having COVID-19 [37]. In this large series we have confirmed that the likelihood to be diagnosed of COVID-19 is higher in MM patients that in the general population.

Finally, we observed that the survival of MM patients decreased in 2020 compared to 2019. Furthermore, we confirmed that the excess mortality in 2020 was higher in MM population than in non-cancer population. This reflect, the major impact of COVID-19 pandemic in vulnerable populations such as MM patients.

In contrast to the non-MM population, patients with MM did not show any improvement in survival results during the second period of the pandemic (June to December 2020), once the first wave of infection (which was the most devastating and stressing for hospital systems in most European countries and the USA) had passed. This finding could be partially explained by a reduced effectiveness of current COVID-19 therapies in this vulnerable MM population -clinical trials for COVID-19 treatments often excluded cancer patients. The lack of improvement in MM patients survival may also be related to the continued adaptations of the health care systems during the pandemic situation.

Overall, a reduced incidence of hematological malignancies has been reported during the first wave of the pandemic, as we have observed in our study, and some studies are addressing the increased risk of getting COVID-19 in these populations [28]. Prior works have also noticed, in line with our findings in MM, that other malignancies such as chronic lymphocytic leukemia or acute leukemias also associate a higher risk of SARS-CoV-2 infections with fatal complications [40–43]. By contrast, chronic myeloid leukemia patients may have not resulted so affected by the pandemic [44–46]. Interestingly, a decrease in the number of hematology-related diagnostic procedures (bone marrow aspirates, flow cytometry assessments, etc) carried out during pandemic period has been reported [47], which could correlate with our observation of lower number of newly diagnosed MM patients during 2020. Since vaccination against SARS-CoV-2 is less effective in hematological neoplasms, these patients might be at risk even after complete vaccination and a specific management different from the overall population may be required to guarantee a better protection [48–51]. Future studies assessing the efficacy of vaccines against SARS-CoV-2 in each particular hematological malignancy and evaluating the impact of the different therapies in the acquisition of COVID-19 immunity will help to optimize the vaccination strategy [52].

There are some study limitations worth mentioning. First, EMR data is subject to data entry errors and data gaps; such as the date of MM diagnosis may not be the actual day of MM diagnosis as it was inferred from the EMR record. Mortality data could be incomplete in some organizations or reported with some delay. We excluded from the survival analysis the data network where out-of-hospital deaths are not tracked consistently. Although data were not centrally curated, H12O MM dedicated datasets were used as a reference for controlling the quality of MM data in all cohorts [19, 20]. One of the strength of this study is the validation of the results in three different data networks around the world that provided very consistent findings for all impact measures. In addition, this study included a large number of patients in the study population of interest contrary to most most MM studies. The large sample size and the use of propensity score matching allowed for more accurate comparisons through controlling for potential factors with clinical and prognostic relevance in an attempt to minimize the risk of bias.

This real-world data global analysis showed that COVID-19 has severely impacted MM patients at different levels. Diagnosis of MM patients was delayed and survival of MM patients was reduced in 2020 compared to 2019. MM patients were more frequently infected with SARS-CoV-2 and had higher excess mortality in 2020 than non-MM patients visiting hospitals. By interrogating large EMR datasets from HCO in Europe and globally, we confirmed that MM patients have been more severely impacted by COVID-19 pandemic than non-MM patients. This study highlights the necessity of extending preventive measures worlwide to protect vulnerable patients from SARS-CoV-2 infection by promoting social distancing and an intensive COVID-19 vaccination strategies.
